# Editorial: Impact of tumor microenvironment on lung cancer

**DOI:** 10.3389/fonc.2023.1136803

**Published:** 2023-01-13

**Authors:** Elena Levantini

**Affiliations:** Institute of Biomedical Technologies, National Research Council (CNR), Area della Ricerca di Pisa, Pisa, Italy

**Keywords:** lung cancer, tumor microenvironment, biomarkers, therapy, molecular crosstalk and signaling

Lung cancer is the leading cause of cancer-related mortality worldwide. In 2022 the Cancer Statistics Center calculated an estimated four new cases and one death per minute from lung cancer solely in the United States (http://cancerstatisticscenter.cancer.org). Lung cancer displays poor survival rates caused by recurrence and metastatic spreading. The anatomical and cellular features of healthy lungs that serve respiratory and defensive purposes, restructure during carcinogenesis to give rise to the tumor microenvironment (TME). This tumor-intermingled “organ” that acts in symbiosis with the cancer cells nourishes their aggressiveness and resistance (1), thus representing a target-rich milieu to hunt for anticancer options.

Despite major efforts the complex pro-tumorigenic interactions occurring within the TME landscape (2, 3) are still substantially uncharted, thus precluding design of thoroughly eradicating strategies. Therefore, research must sturdily focus on discovering biomarkers to facilitate early diagnoses, improve clinical predictions and stratification parameters, as well as identifying new avenues to target cancer cells vulnerabilities and to modulate the pro-tumorigenic TME. Within the TME, the immune infiltrating system and the stromal cells have emerged as therapeutic, diagnostic and prognostic tools in NSCLC (4–6). *In this Research Topic*, several novel biomarker signatures were presented, contributing to the ever-growing puzzle describing the entire complexity of pulmonary TMEs. A 14-TME-related gene signature was identified to exhibit significant prognostic potential for patients affected by malignant pleural mesothelioma (MPMs), a rare but highly aggressive thoracic malignancy [Xu et al.]. Similarly, analyses from 450 NSCLC cases identified key immune cell types associated with recurrence of stage IA-B NSCLC within 40 months after surgical resection, contributing to develop an immune-related gene panel that predicts relapse-free survival (RFS) [Wang et al.]. Focusing on the immune-related genes differentially expressed in lung adenocarcinomas (LuADs) displaying high and low immune/stromal scores, *Colony-stimulating factor 2 receptor beta* (*CSF2RB*) was identified as a hub gene within LuAD-TMEs [Zhu et al.]. Low *CSF2RB* expression was associated with poor survival and *CSF2RB* was identified as an independent risk factor for prognosis, independent of whether patients received chemotherapy or radiotherapy. More importantly, a high expression of CSF2RB was related to early T, N, and clinical stages. Further studies are expected to elucidate the tumor-suppressor function elicited by CSF2RB in pulmonary TMEs, testing the attractive hypothesis that CSF2RB, the shared subunit of interleukin 3 (IL3), GM-CSF, and IL5 receptor (7), is directly involved in modulating immune infiltration [Zhu et al.]. Another study revolved around the ceramide-pathway, that is emerging in cancer research for its potential roles in proliferation and cell-to-cell communication (8). A signature composed of three specific ceramide-regulated genes (among the 22 comprising the pathway) was shown to be more highly expressed in LuAD than in normal-adjacent tissues, representing a prognostic risk signature able to classify patients into high- and low risk subgroups [Zhang et al.]. In lung squamous carcinoma (LuSC), immune infiltration scores identified four subtypes, namely, subtypes 1 and 2 (mixed type), subtype 3 (tumor enriched), and subtype 4 (immune enriched, fibrotic), all displaying distinct prognoses [Yin et al.]. Subtype 3 had the best overall survival (OS), while subtype 4 had the worst. A risk model was developed, adopting 7 immune-related differentially expressed genes (DEGs) between subtype 4 and subtype 3, that proved able to predict OS rates in multiple validation datasets. Among these genes, Formyl Peptide Receptor 1 (FPR1), an important regulator of neutrophil recruitment (9), was able to increase migration and invasion abilities of lung squamous cancer cells upon its activation. In most cancers, high neutrophil infiltration is associated with poor prognosis (10, 11); however neutrophils can also mediate antitumor responses (12), thus requiring further consideration is given to FPR1 in the preclinical arena to pave the way for improved strategies for lung cancer management.

Stage-dependent immune cell infiltrations have prognostic utility, and specific TME states are being considered as potential biomarkers to determine stage, clinical outcome and therapeutic responses (13). The innate and adaptive immune cells within the pulmonary TME hold both tumor-promoting and tumor-suppressing activities, which may also predict clinical outcome (14, 15) and treatment response (16, 17).

*In this Research Topic*, the immune cell composition of paired NSCLC specimens and adjacent normal lung tissues from patients undergoing radical resection, revealed defined infiltration patters. Compared with adjacent normal lung tissues, an increased proportion of CD4^+^ T cell subtypes, Tregs and B cells was observed in tumor samples, with reduced frequencies of myeloid cell populations [Su et al.]. There was no significant increase in total CD8^+^ T cells, but both PD1^+^ and CD38^+^ CD8^+^ T cells were enriched in tumors, displaying association with tumor size. A high proportion of CD8^+^ T cells and a low percentage of PD1^+^ CD8^+^ T cells were associated with better survival in stage II and III patients, whereas a low frequency of CD38^+^ CD8^+^ T cells was associated with better survival in all patients and identified as an independent prognostic factor.

Identifying guidelines to characterize the immune component of pulmonary TMEs is crucial, given the applicability of recent immunotherapy strategies. However, additional parameters able to molecularly describe the totality of the TME, composed by tumor cells, extracellular matrix, endothelial, fibroblasts and immune cells, will eventually flow into future multiscale profiling approaches to build up patient-specific TME atlases, to guide physicians to choose more efficient therapeutic options. To examine the crosstalk between vessel endothelial cells (ECs) and metastatic tumor cells, experiments performed by conditioning lung cancer cells with human brain microvascular endothelial cells (HBMECs), showed the homeobox-containing gene *HOXB9* may be involved in the process. Its upregulation upon exposure to ECs, fits with the observation that higher expression of HOXB9 is associated with poor OS in lung cancer patients [Wei et al.].

Finally, in order to introduce all the newly-discovered parameters describing TME dynamics, we discuss the relevance of tumor budding (TB), namely the presence of individual cells or small clusters of tumor cells at the invasive front of tumors, as potential prognostic factor in lung cancer. TB was originally described in colon adenocarcinomas (18), and the comprehensive systematic meta-analysis presented here confirms it as predictive of poor prognosis in both LuAD and LuSC. Smoking was one of the most important findings associated with higher TB, suggesting it may be activated through the EMT pathway. Importantly, the need to standardize TB assessment criteria in the clinics is discussed, to universally homologate the prognostic scores [Thakur et al.].

This roundup concludes the topics discussed *in this Research Topic*, which is expected *to be continued* with efforts from the worldwide preclinical and clinical community. At the present stage, we should also start considering an extra level of complexity, in that the major heterogeneity displayed by tumors and their intertwined TMEs (19, 20) may also be patient-specific, thus representing a major obstacle in identifying the relevant targets to adopt each time. Historically, studies have been addressing the composition of the TME by flow cytometry or immunohistochemistry, or by bulk genomic/transcriptomic analyses. It is now time to start focusing on single cell interactions, and the dense jungle/tangle they build up around tumors, following altered and dysregulated molecular cues, in order to conceive upgraded weapons against lung cancer by adopting enhanced and *ad hoc* personalized medicine tools.

## Author contributions

The author confirms being the sole contributor of this work and has approved it for publication.
